# Towards Self-Assembling 3D-Printed Shapes Through Βiomimetic Μechanical Interlocking

**DOI:** 10.3390/biomimetics10060400

**Published:** 2025-06-13

**Authors:** Tino Marte, Savvas Koltsakidis, Thomas Profitiliotis, Emmanouil Tzimtzimis, Dimitrios Tzetzis

**Affiliations:** 1 Digital Manufacturing and Materials Characterization Laboratory, School of Science and Technology, International Hellenic University, 57001 Thessaloniki, Greece; 2 Biomimetics-Innovation-Centre (B-I-C), School of Nature and Engineering, Bremen University of Applied Sciences, 28199 Bremen, Germany

**Keywords:** self-assembly, interlocking, biomimetic design, stereolithography

## Abstract

While early studies on macroscopic self-assembly peaked in the late 20th century, recent research continues to explore and expand the field’s potential through innovative materials and external control strategies. To harness this potential, a unit cell was designed and 3D-printed that could form a face-centered cubic lattice and stabilize it through a biomimetic mechanism for mechanical interlocking. The wing coupling structures of the brown marmorated stink bug were examined under a scanning electron microscope to be used as a source of bio-inspiration for the interlocking mechanism. A total of 20 unit cells were studied in five different self-assembly processes and in different compression scenarios. A maximum average of 34% of unit cells remained stable, and 20% were mechanically interlocked after self-assembly tests. The compression tests performed on a single unit cell revealed that the cell can withstand forces up to 1000 N without any plastic deformation. Pyramid configurations from 5-unit cells were manually assembled and assessed in compression tests. They showed an average compression force of 294 N. As the first study focused on self-assembly through mechanical interlocking, further studies that change the unit cell production and self-assembly processes are expected to improve upon these results.

## 1. Introduction

The basic building blocks of life have no conscious actor who assembles them. A variety of substances need to spontaneously order specialized proteins and membranes to emerge [1]. This spontaneous ordering into a functional structure is termed self-assembly [2]. Humans are attempting to replicate this feat for technological applications [3]. The goal from the beginning was man-made self-assembly on a micro- or nano-scale [4,5]. Macroscopic self-assembly was used as a stepping stone on the path to that goal [6,7]. An intentional focus on the self-assembly of structures on a macroscopic scale could lead to the emergence of macroscopic technologies that automatically construct and repair from smaller components [8].

While the initial surge of interest in macroscopic self-assembly took place in the late 20th and early 21st centuries, more recent investigations indicate that the field has continued to evolve in various directions. For example, studies have examined the programmable organization of millimeter-scale components using magnetic interactions [9] and the capillary-driven structuring of macroscopic elements [5]. In addition, approaches involving robotics and active materials have broadened the scope of related research, even if these systems fall outside the strict definitions of self-assembly [10]. Thus, while early work laid the foundation, contemporary research continues to advance the field, warranting a more nuanced discussion of its ongoing developments.

One phenomenon leads to favorable circumstances for the self-assembly of macroscopic components. Separate solids in a fluid, such as air or water, have the tendency to spontaneously order in dense arrangements [11]. This decrease in entropy for solids has been hypothesized to occur due to an increase in entropy in the surrounding fluid [12]. If the unit cells are densely packed, self-assembly will occur on its own without the need for any intentional action.

For a self-assembled material to withstand loads, forces that keep its components ordered need to be present. Previous studies have used a variety of bonding mechanisms to stabilize self-assembled structures. Gracias et al. [13] used a molten metallic alloy to assist with assembly through capillary forces and solidified it after, soldering shapes together. Terfort et al. [14] used a hydrophobic liquid film of adhesive on surfaces that assisted during assembly and could later be polymerized. Each required special equipment and a controlled environment along with a separate bonding process after self-assembly. If self-assembly can occur only through the intrinsic properties of the unit cell, it eliminates the need for any external additions such as adhesives. Mechanical interlocking can serve as a bonding mechanism that occurs due to the intrinsic property of geometry.

An interlocking mechanism that engages during a self-assembly process without also disengaging at the same rate is a problem unique to this proposed application. Biomimetics is an interdisciplinary approach to solving technical problems through the abstraction, transfer and application of knowledge gained from biological models [15]. Insects have developed a variety of ways to easily and securely connect one structure to another, including mechanical interlocking [16]. A biomimetic approach can be deployed to harness the millions of years of evolutionary development in insects to conceive the needed mechanism.

Additive manufacturing is a technology that is seeing increased scientific interest and development [17]. Because it can produce intricate shapes in low volumes with minimum investment, it creates design opportunities for research that were previously inaccessible [18]. It offers advantages such as excellent dimensional accuracy and surface finish, making it suitable for various applications including dentistry and lightweight lattice structures [19,20]. SLA encompasses different methods like laser-based SLA, digital light processing (DLP) and advanced techniques such as two-photon polymerization (TPP) and continuous liquid interface production (CLIP) [21]. Recent developments in light excitation modes, particularly the transition from one-photon to two-photon mechanisms, have expanded material possibilities and enabled true 3D printing without layering [22]. However, SLA also has drawbacks, including high costs and limited material options compared to other am3D printing technologies [19,21]. For the design of novel concepts such as unit cells that can self-assemble through mechanical interlocking, it provides advantages such as rapid prototyping [23]. The stereolithography used in modern resin printers (SLA) offers the high resolution and accuracy required for creating small interlocking parts [24].

Interlocking mechanisms in 3D printing have gained attention for their ability to create strong connections between parts without additional fasteners. Researchers have developed various approaches to designing and fabricating interlocking structures. Song et al. [25] proposed a voxelization-based method to partition 3D models into interlocking parts, enabling the assembly of large objects. Alemanno et al. [26] introduced a technique to decompose 3D shapes into height field pieces with interlocking mechanisms, suitable for cultural heritage replicas. Gloyer et al. [27] designed a snapping interlocking meta-surface mechanism using flexible materials, demonstrating strong planar surface connections. Hamilton et al. [24] optimized micro-structured adhesive joints through finite element modeling and 3D printing, finding that mechanical interlocking accounts for most of the joint’s load-carrying capacity. These studies highlight the potential of interlocking mechanisms to enhance 3D-printed objects’ assembly, strength and functionality across various applications.

**Figure 1:**
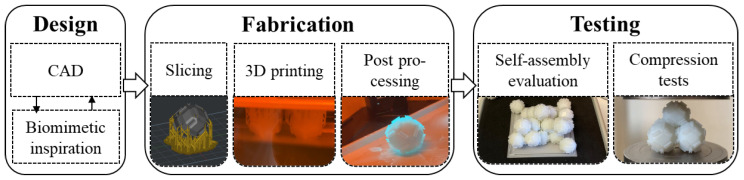
Methodology chart depicting general structure of all processes performed in Materials and Methods Section. Photo documentation of production and testing is depicted underneath each corresponding step.

This work aims to utilize the aforementioned principles by designing a macroscopic unit cell that can form a densely packed lattice. The spontaneous ordering of the unit cells in a fluid may result in the self-assembly of the unit cells in the desired configuration. Such a lattice could intrinsically remain stable through biomimetic mechanical interlocking and withstand the loads needed for practical applications. To verify these hypothesized properties, a unit cell was biomimetically designed based on interlocking mechanisms found in insects and produced using 3D printing. The current work introduces a novel approach by applying the concept of mechanical interlocking to a densely packed, three-dimensional lattice composed of rhomboidal dodecahedron (RD) unit cells. A key innovation of this study lies in the geometric adaptation of the RD, a polyhedron well-suited for dense packing but inherently lacking interlocking features. By chamfering every edge of the RD, we created voids that enable three distinct shapes to interlock at a single junction. This work therefore extends current knowledge by demonstrating a macroscopic unit cell design that supports biomimetic interlocking and self-assembly through 3D printing, which has not been addressed in prior interlocking mechanism research. The methodology is summarized in Figure 1. The compression behavior of a single unit cell was investigated, while the maximum potential stability was examined by performing simple compression tests on pyramid configurations manually assembled from the same unit cells. Self-assembly processes were performed on 20 unit cells and evaluated based on the percentage of stable and interlocked unit cells.

## 2. Materials and Methods

### 2.1. Design 

The design of the unit cell was conducted in Solidworks^®^ Edition 2023 SP2.1 (Dassault Systèmes SolidWorks Corp., Waltham, MA, USA). To decide which geometry the unit cells should be based on, the configuration of its spontaneous ordering must be understood. Damasceno et al. [28] simulated the self-assembly of polyhedra and the resulting lattice structure. Geometries with equal sides and a high level of symmetry were the criteria determined as favorable for self-assembly. They represent the possible orientations for each unit cell to be able to integrate into an assembling lattice. Desirable lattices are face-centered cubic and body-centered cubic, which have been trrfound to have the lowest anisotropy out of all cubic lattices [29]. One polyhedron that possesses all beneficial properties mentioned previously is the rhomboidal dodecahedron (RD). Thus, it was chosen as the base for the unit cell of a self-assembling material.

**Figure 2:**
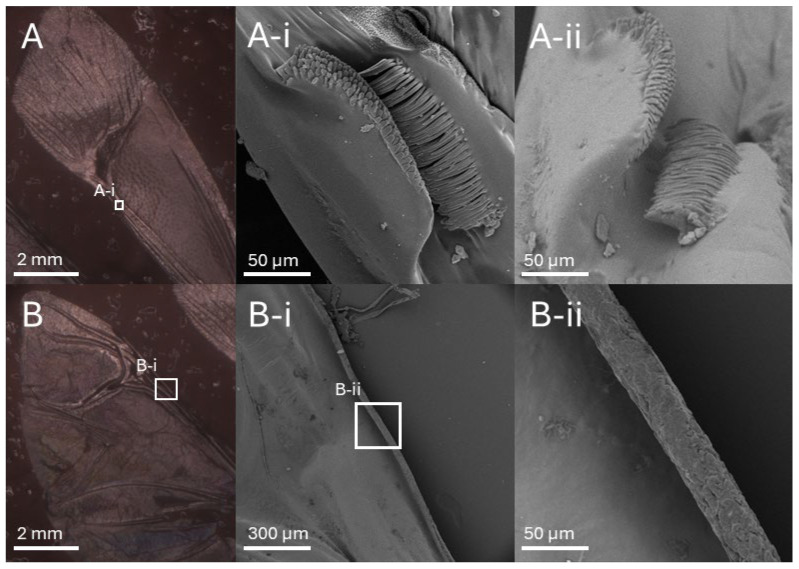
Microscopic images showing the wing interlocking mechanism of the brown marmorated stink bug. In (**A**) the fore wing is depicted and in (**A-i**) and (**A-ii**) its interlocking structure from 2 different angles. It consists of an upper protrusion that has short microtrichia on top and a lower hooked structure formed by many longer microtrichia. (**B**) depicts the hind wing and (**B-i**) and (**B-ii**) its part of the interlocking mechanism at two different magnifications. Visible is the rolled-up leading edge of the wing coated in flattened microtrichia. It acts as a counter-oriented hook to the one found on the fore wing during the engagement of the mechanism.

An RD does not inherently possess any mechanism for mechanical interlocking and needs to be adapted. A lattice of RDs is densely packed and offers no space to include interlocking structures. Chamfering every edge of the RD creates this space while allowing for the interlocking of three different shapes in one junction. For the biomimetic approach to the design of the interlocking structures, the mechanical interlocking mechanisms in insects must be analyzed. Ma et al. [30] reported on a unique wing coupling mechanism in the green stink bug. To obtain a deeper understanding of these mechanisms found in stink bugs, the wings of a brown marmorated stink bug were investigated. The animal was taken from its natural habitat in Thessaloniki, Greece, and killed by being exposed to a cloth doused in isopropyl alcohol. The wings were removed from the body and dried overnight in a drying oven at 35 °C. They were sputter-coated with gold. The prepared specimens were then examined inside of a scanning electron microscope (Phenom ProX G5, Thermo Fisher Scientific Inc., Waltham, MA, USA). The resulting images can be found in Figure 2.

The mechanism of this stink bug was found to be nearly identical to the mechanism described by Ma et al. [30]. To mechanically interlock, a rolled-up edge of the hind wing acts as a hook and engages with a counter-oriented hooked structure on the fore wing.

**Figure 3:**
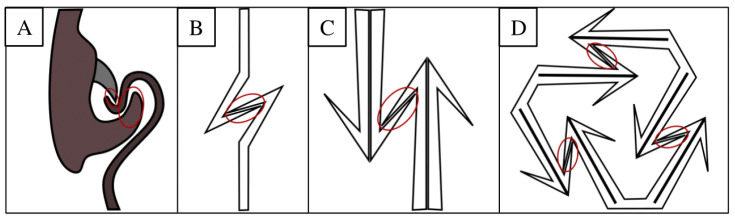
An iterative biomimetic design of the interlocking mechanism. Interlocked surfaces are circled in red. (**A**) A schematic of the cross-section of the engaged wing coupling structures found in Figure 2 is depicted. Shown in (**B**) is the simplified abstraction of that mechanism as two counter-oriented hooks. In (**C**), an adaptation of the previous design is depicted so that it may engage in multiple orientations by mirroring the hooks to form an arrow-like shape. (**D**) depicts an adapted version of (**C**) so that it may interlock in a stable configuration of three, instead of two. This is performed by rotating the arrow to one side by 60° and mirroring it in the other.

Another wing coupling mechanism using the same basic principle is found in wasps and described by Eraghi et al. [16]. May beetles and lady bugs have developed forms of mechanical interlocking through hooks to stabilize their elytra in their closed positions [31,32]. Though these examples have a range of applications, all share common elements. There are two hooked protrusions in opposite orientations that engage, preventing disengagement under tension. Similar mechanisms have independently developed in multiple species, showcasing the effectiveness of this form of mechanical interlocking. Thus, they are chosen as sources of bio-inspiration. Because these mechanisms are dependent on the orientation of the hooks, their engagement needs to be guided for interlocking to be successful. For their application in self-assembly, they need to be abstracted and adapted (see Figure 3).

**Figure 4:**
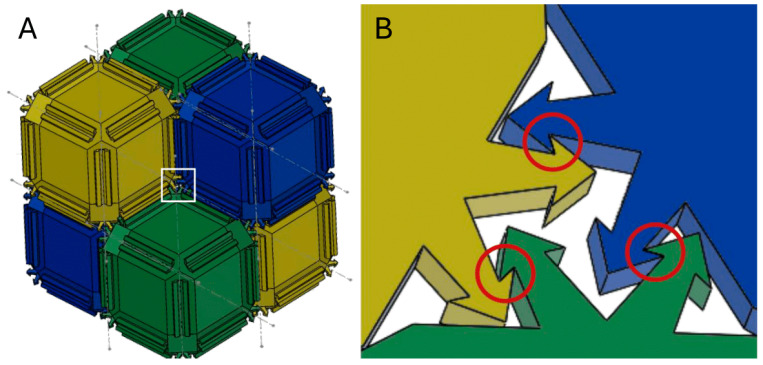
The design of the proposed unit cell. Depicted in (**A**) is the CAD assembly of 6 identical unit cells colored in yellow, blue and green for visual clarity. Notable are the chamfered edges with hooked protrusions placed on top. In (**B**) a schematic of 3 mechanically interlocked protrusions is depicted, similar to the one highlighted in a white rectangle in (**A**). The interlocking regions highlighted in red circles use hook-like ridges in opposite orientation, as an analog to mechanical interlocking in insects such as the brown marmorated stink bug.

The proposed unit cell is designed based on the aforementioned properties. It is based on the shape of an RD with chamfered edges. A biomimetic mechanism for mechanical interlocking based on the principle of counter-oriented hooks is added on the bevels that were generated by chamfering (see Figure 4A). Many unit cells can form a densely packed lattice, where each shape is mechanically interlocked with two others on each of its edges (see Figure 4B).

### 2.2. Fabrication

**Figure 5:**
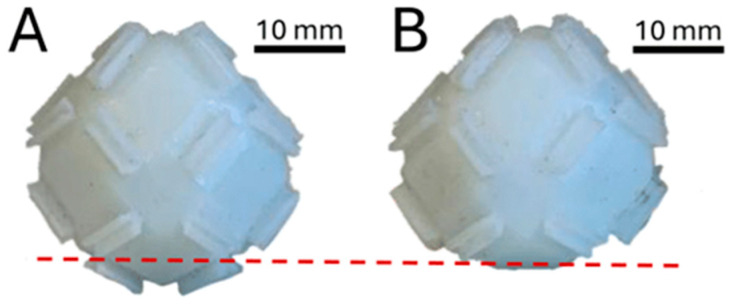
Photos of the unit cell shape and size to be produced. Depicted in (**A**) is one of the 20 regular unit cells. In (**B**) one of the 9 trimmed unit cells is depicted. The cut on the bottom, shown as a dashed line in red, leads to a flat surface that makes it possible to form a stable base.

All unit cells were produced with Liquid Crystal Display (LCD)-Based Masked Stereolithography (MSLA) on a resin printer (HALOT-MAGE S 14K, Creality, Shenzhen, China) with ABS-like resin (Hard-Tough Resin, eSUN^®^, Shenzhen, China). The print settings can be found in Table 1. The prints measure a maximum radius of 31 mm. A total of 29 cells were produced this way. Nine of these cells were trimmed by 5 mm down to 26 mm perpendicular to the axis of the maximum radius with a precision cut-off machine (Minitom, Struers, Copenhangen, Denmark) (see Figure 5).

### 2.3. Compression Tests

Experiments were conducted using a universal testing machine (Testometric M500-50AT, Testometric, Lincoln, UK) at a constant testing speed. The machine was equipped with a load cell capable of handling up to 50 kN. A singular unit cell was assessed with a speed of 5 mm/s. The assembly behavior was tested on a manually assembled pyramid of 5 unit cells at velocities of 5 mm/s and 20 mm/s. This configuration was chosen because it was commonly observed during self-assembly.

### 2.4. Self-Assembly Tests

**Figure 6:**
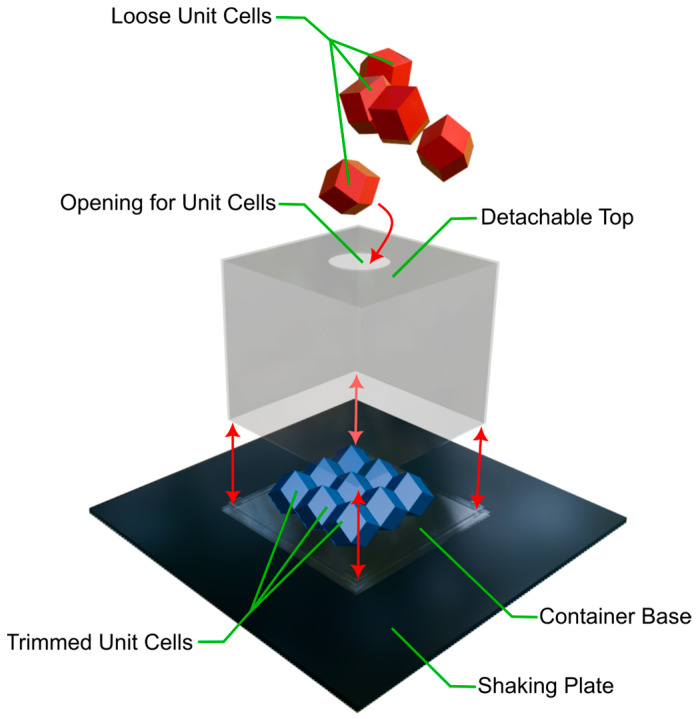
A simplified model of the setup used for the self-assembly experiment. Arrows depict the movements of parts during setup.

To test for self-assembly, an orbital shaker (PSU-10i, bioSan, Riga, Latvia), a custom cubic container with a detachable top and all 29 unit cells were used. The 9 trimmed unit cells were pre-assembled in a 3 × 3 × 1 configuration, and the trimmed, flat sides were attached to the container base with adhesive. The closed container was placed on the shaking plate and the remaining, loose unit cells fed into the opening on the top of the container one by one. Then the container was securely attached to the plate with adhesive tape, and the shaking plate started. A variety of settings for shaking were used and compared (see Table 2). Afterwards the top was detached and all cells that remained stable counted. Lastly the stable configuration was manually disassembled and the number of interlocking cells noted. After all tests, a control was conducted that followed the same process but never turned on the shaking plate. The detachment of the top, stable configuration and disassembly were graphically documented in the form of a video. The setup is depicted in Figure 6.

## 3. Results

### 3.1. Interlocking Mechanism

The produced unit cells could be printed with enough accuracy to allow for mechanical interlocking to occur. The thickness of interlocking structures was found to vary depending on the printing orientation. This is because SLA printing can result in variations in structural thickness depending on the printing orientation due to differences in resolution across the X, Y and Z axes. Typically, SLA printers offer the highest resolution in the Z axis (vertical direction), controlled by the layer height, which can be as fine as a few microns. In contrast, the resolution in the X and Y axes is limited by the laser spot size and the optical system, which can result in less precision, particularly in lateral dimensions. Moreover, printing orientation significantly influences the formation of overhangs and unsupported features. When interlocking elements are printed in orientations that produce overhangs, the presence of unsupported layers can lead to resin sagging or inadequate curing, which affects the final thickness and shape accuracy. In such cases, features may appear thicker or less defined than intended due to gravitational effects and the accumulation of excess resin during the curing process.

**Figure 7:**
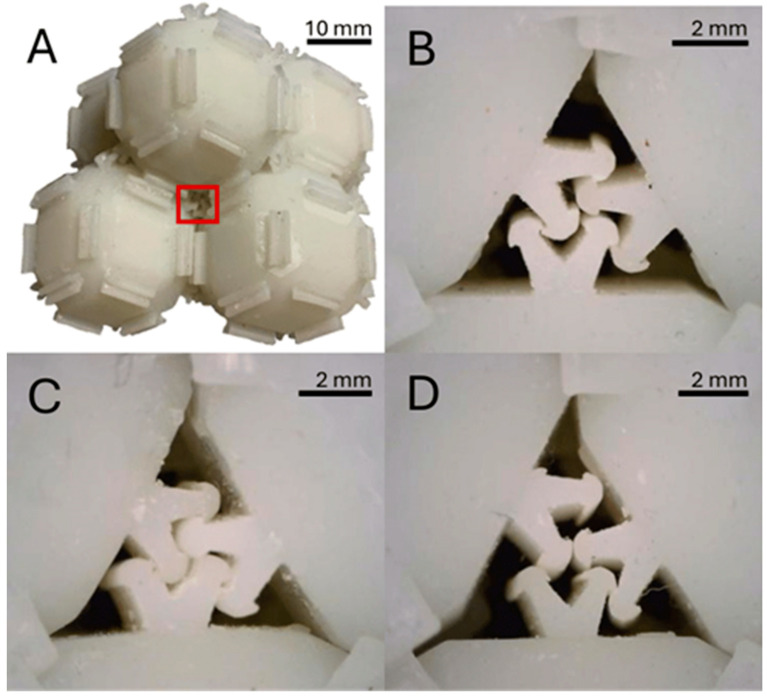
Photos of the produced unit cells. In A, the pyramid configuration that was commonly observed in self-assembly and chosen for material assessment is depicted. (**A**) A red rectangle highlights a junction like the one shown in (**B**–**D**). Depicted in (**B**) is a junction on edges that were printed in the same orientation and show a comparatively low thickness of protrusions. In (**C**) the same is depicted but with edges from a printing orientation that shows a comparatively high thickness of protrusions. In both cases, all hook-like ridges can engage, and the sides sit flush. In (**D**) edges printed in different orientations with protrusions of comparatively high variable thickness are depicted. It is notable that the top left protrusion has no space to integrate into the configuration, and interlocking is hindered. The result is a gap between sides visible in the bottom left corner. This configuration represents a defect in the lattice, as it hinders the proper engagement of adjacent unit cells.

When thickness is similar between interlocking structures, interlocking could occur (see Figure 7B,C). If the thickness varied between protrusions, complete engagement is prevented, and the integration of further shapes into the lattice may be hindered (see Figure 7D). An improvement in the production focused on consistency could lead to a higher degree of interlocking during self-assembly. This may also improve the strength of interlocking and lead to the higher compression strength of an assembly. Higher accuracy in production presents a promising avenue for future studies to pursue.

### 3.2. Compression Results

**Figure 8:**
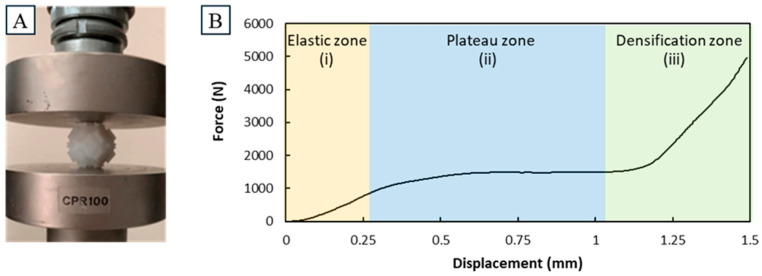
Simple compression tests on a single unit cell. Depicted in (**A**) is the setup of the single unit cells between the compression attachments. In (**B**) the force–displacement curve at a compression speed of 5 mm/s is depicted. Notable is the elastic zone up to 1000 N followed by a plateau zone around 1500 N. The end of the curve shows a densification zone with a comparatively high slope to the initial one.

The behavior of a singular unit cell in a simple compression test is shown with an indicative force–displacement curve in Figure 8. Three different sections can be observed: 1. an elastic zone with a comparatively shallow incline, 2. a plateau zone, and 3. a densification zone with a comparatively steep incline. A plateau was observed there, which is likely the result of the small protrusion yielding, until giving way to the main body, where forces would rise again. The visual changes observed in the unit cell after compression further support this, as the main body remained intact, while the protrusions had yielded up to being flush with the compressed side of the main body. This behavior could first be observed at around 1000 N, far above the maximum measured forces in the compression of the pyramid assemblies. 

**Figure 9:**
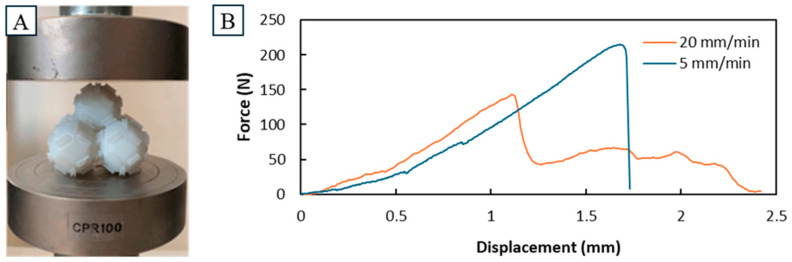
Simple compression tests on a pyramid assembly. Depicted in (**A**) is the setup of the pyramid assembly between the compression attachments. In (**B**) the force–displacement curves at compression speeds of 5 mm/s and 20 mm/s are depicted. The curves end when the unit cells had fully disassembled from the load, seen at the sudden drop in the measured forces to near zero. The compression strengths measured were higher at the lower compression speed.

To showcase the behavior of the pyramid assemblies in the simple compression tests, two indicative force–displacement curves at the two different compression speeds are depicted in Figure 9. Both show a high degree of non-linearity. At the higher compression speed, lower compression strengths were measured at 115 N and 150 N. In comparison, the lower compression speed showed compression strengths ranging from 208 N to 438 N with an average of 294 N (n = 4). Even in this early stage of development, this indicates that the compression strengths can achieve heights adequate for practical use in a variety of applications. Finally, once the peak strength was reached, the physical displacement of the cells was observed. This displacement appeared more significant during the low-speed test, likely due to the higher force generated. A more substantial dislocation of the cells, in turn, led to a sharp decline in the force curve, as the testing machine encountered minimal resistance, primarily because the individual cell structures have a notable lower height than the assembly.

To further clarify the role of interlocking structures, their contribution lies in controlling how the assembly deforms under compression through mechanisms such as slipping, shifting and temporary disengagement. These behaviors are not present in the singular unit cell, indicating that they arise from the collective interaction of interlocked shapes. This highlights the importance of the interlocking geometry in shaping the mechanical response of the overall structure, beyond what can be explained by material properties alone.

### 3.3. Self-Assembly Results

**Figure 10:**
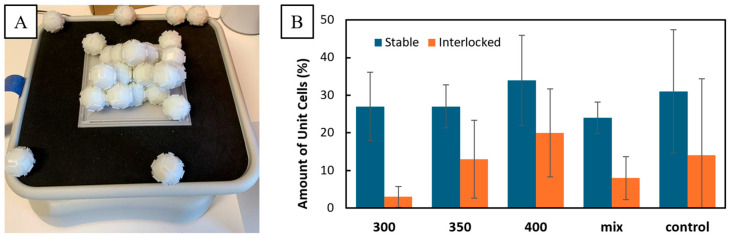
Self-assembly experiments. An example trial showcasing multiple frequently observed configurations is depicted in (**A**). On top of the shaking plate, the base with 11 stably assembled shapes is visible. Six cells on the outer edges of the shaking plate, two in the upper corners and one in the lower right corner of the base are loose and have not assembled. The assembled shapes form a pyramid formation in the middle. The others assembled on the sides around the pyramid. In (**B**) a chart depicting the average percentage of unit cells (n = 5) remaining stable or interlocked after the removal of the container top for different self-assembly processes is depicted. Error bars correspond to standard deviation. With increasing rpm, the average interlocking percentage increased. Stability does not show a clear pattern but still has the highest average percentage at the highest rpm with 34% of the unit cells remaining stable and 20% being interlocked. The control that was not shaken shows high interlocking and stability percentages with high standard deviations.

In Figure 10B the percentage of unit cells showing interlocking or stability in shapes for all different shaking speeds are depicted. Interlocking seemed to be proportional to the rotational speed of the orbital shaker used during self-assembly. Both interlocking and stable unit cells could be observed even when there was no shaking. This was seen in a control that measured immediately after the initial deployment of unit cells through a central opening on the top of the container. In fact, the measurement with the most stable and interlocked unit cells was taken with this setup with 60% of shapes remaining stable and 50% being interlocked. This resulted in the high standard deviations of the control, as the other measurements were comparatively low. The fact that even with no shaking a high measurement could be recorded means that the self-assembly of loose unit cells into an interlocked lattice could be achieved in a short time and with minimal movement. Shaking at 300 rpm for 6 min seems to even have had a negative impact on interlocking compared to no shaking. Generally, shaking had little impact on stability, as it only deviates slightly between setups. This brings into question the practicality of a rotary shaking motion for the self-assembly of the 3D-printed unit cells tested here. This could be because of the gravity that constantly affects the shapes during shaking, pushing them downward and making them settle into positions that are highly stable with the container but unstable without it. Without a force counteracting gravity, these states may rarely be disrupted and could hinder the self-assembly of adjacent cells. With the control showing that vertical initial momentum through gravity could yield a high degree of stability and interlocking, the movement of the cells vertically seems to be the driving factor for the self-assembly observed in the experiments. This may be the reason why the highest rotational speed led to the highest average stability and interlocking, as these high speeds could result in vertical momentum from the small impacts between unit cells. Further experiments with different movement directions may be needed to more closely understand the self-assembly processes reliant on mechanical interlocking.

## 4. Conclusions

A unit cell that can form the densest packed face-centered cubic lattice with a biomimetic interlocking mechanism was designed. Various unit cells were 3D-printed through Liquid Crystal Display (LCD)-Based Masked Stereolithography (MSLA). A pyramid assembly made from five of these unit cells could remain stable when interlocked and withstand a compression load up to 438 N at a compression speed of 5 mm/s. At a higher compression speed of 20 mm/s, the highest measured compression strength was 150 N. Imperfections based on the printing orientation of edges could be observed, leading to the diminished functionality of the interlocking mechanism. Future studies can focus on improving this aspect, which could produce general improvements for all recorded data in this study. Twenty unit cells were tested in self-assembly processes fueled by the movement of a rotary shaker and evaluated based on the amount of stable or interlocked cells. The highest recorded average percentage of stability and interlocking among unit cells was at the highest tested rotational speed with 34% of unit cells being stable and 20% showing interlocking. A control with no shaking was conducted and recorded the highest singular measurement with 60% stability and a 50% interlocking percentage. This means that the rotational movement may not have been the main driver of the observed self-assembly, and vertical shaking could yield higher percentages of interlocking and stability. Studies focused on the influence of different types of movement on the self-assembly processes reliant on mechanical interlocking are needed for more concrete conclusions.

## Figures and Tables

**Table 1:** Print settings used to produce unit cells.

Initial Exposure	Exposure Time	Rising Height	Motor Speed	Light-Off Delay
30 s	2.8 s	8 mm	5 mm/s	6 s

**Table 2:** Self-assembly setups for self-assembly with the settings of the orbital shaker. One setup used all previous speeds decreasing from the highest to the lowest for 2 min each. A control was conducted that did not shake the plate. All others shook for 6 min at a constant speed.

Test	Time (min)	Speed (rpm)
300	6	300
350	6	350
400	6	400
Mix	2/2/2	400/350/300
Control	0	0

## Data Availability

The data that support the findings of this study are available from the corresponding authors upon request.
